# Energy-Dependent Rash Triggered by Bilateral Electroconvulsive Therapy: A Rare Case Report

**DOI:** 10.1192/j.eurpsy.2026.11743

**Published:** 2026-07-31

**Authors:** A. Uzturk, I. H. Karakus, S. S. Kirlioglu Balcioglu

**Affiliations:** Department of Psychiatry, Basaksehir Cam and Sakura City Hospital, Istanbul, Türkiye

## Abstract

**Introduction:**

Electroconvulsive therapy (ECT) induces controlled seizures via electrical stimulation.It is used in severe or treatment-resistant depression, mania, schizophrenia, and schizoaffective disorder(American Psychiatric Association, 2025).Dermatological reactions to ECT are exceedingly rare.Kellner et al. (2015) reported ipsilateral hemifacial erythema during right unilateral ECT.To date, there have been no documented cases of erythematous rashes associated with bilateral ECT.

**Objectives:**

We report a rare case of energy-dependent erythematous rash induced by bilateral temporal ECT.

**Methods:**

A 29 year old woman with treatment-resistant paranoid schizophrenia was hospitalized for aggression, disorganized behavior, seeing images, and hearing voices.She had no history of ECT.On admission, she exhibited decreased self-care, disorganized thinking, irritability, auditory and visual hallucinations, persecutory and mystical delusions, impaired insight. Laboratory tests, urine toxicology, and cranial imaging were normal.

**Results:**

Due to persistent psychotic symptoms despite adequate antipsychotic therapy, bilateral ECT was initiated.Over seven sessions, erythematous rashes appeared on the neck and chest within seconds of electrical stimulation. The rashes resolved within minutes following administration of corticosteroids and antihistamines. Allergy testing for anesthetic agents and latex (latex-specific IgE) yielded negative results. A notable pattern emerged; as the ECT stimulus energy increased, the rash became more pronounced, requiring higher doses or combined regimens of steroids and antihistamines (Table 1).No hemodynamic instability was observed, and ECT was otherwise well tolerated. Marked clinical improvement was noted, with PANSS scores improving from 116 to 75 and BPRS scores from 33 to 18.The patient regained partial insight and was discharged.

**Image 1:**
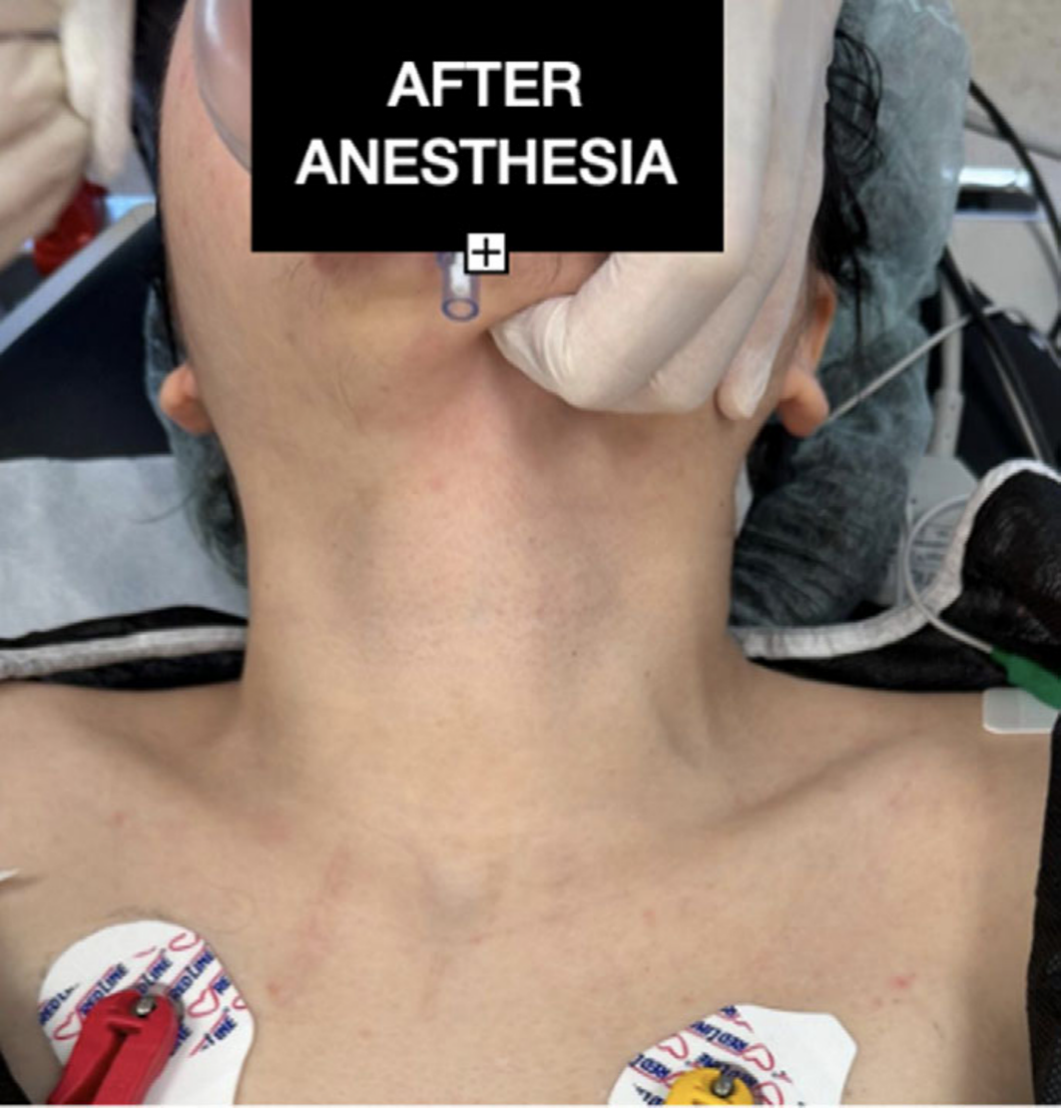

**Image 2:**
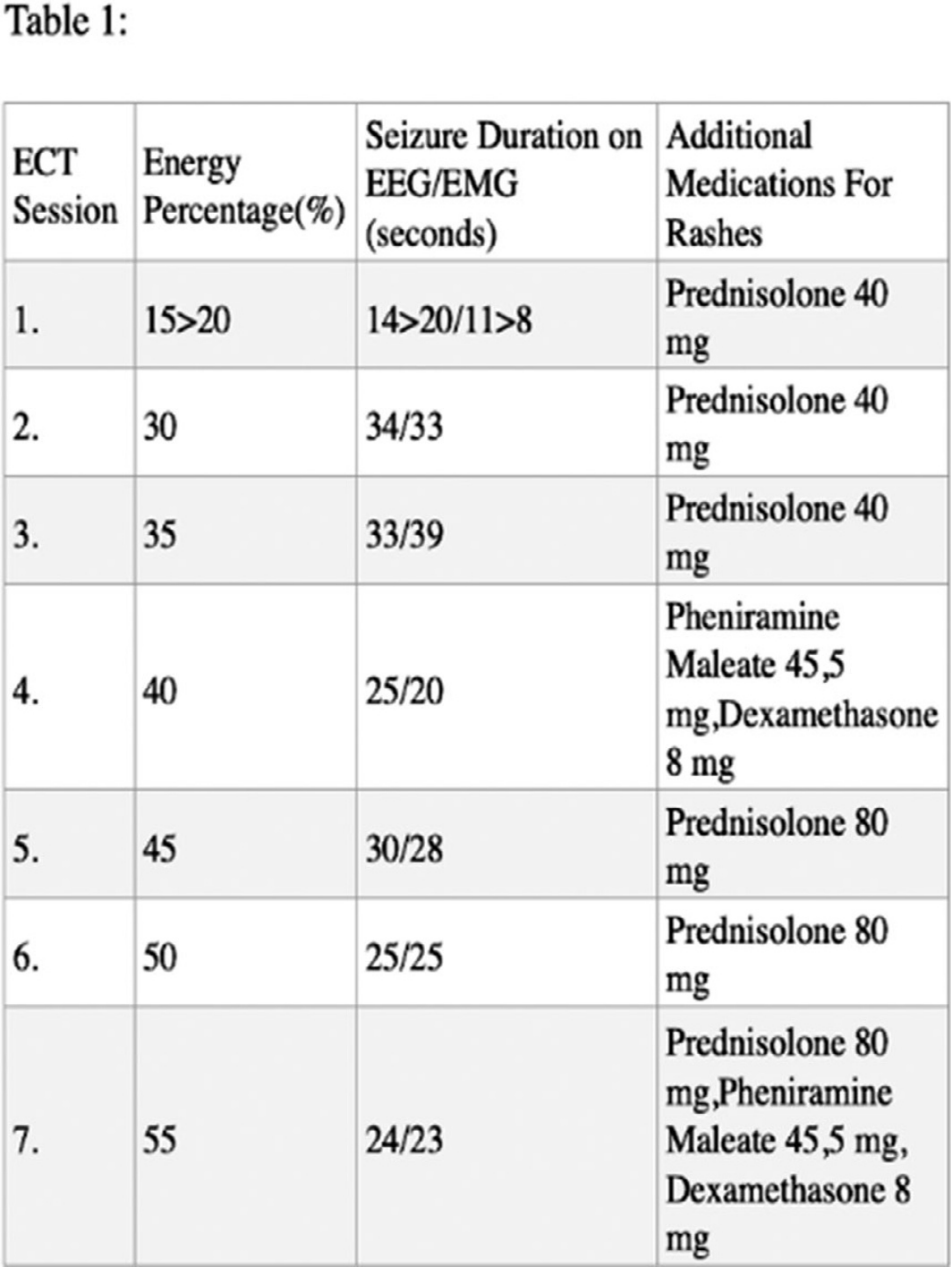
Image 2: Long description.

**Image 3:**
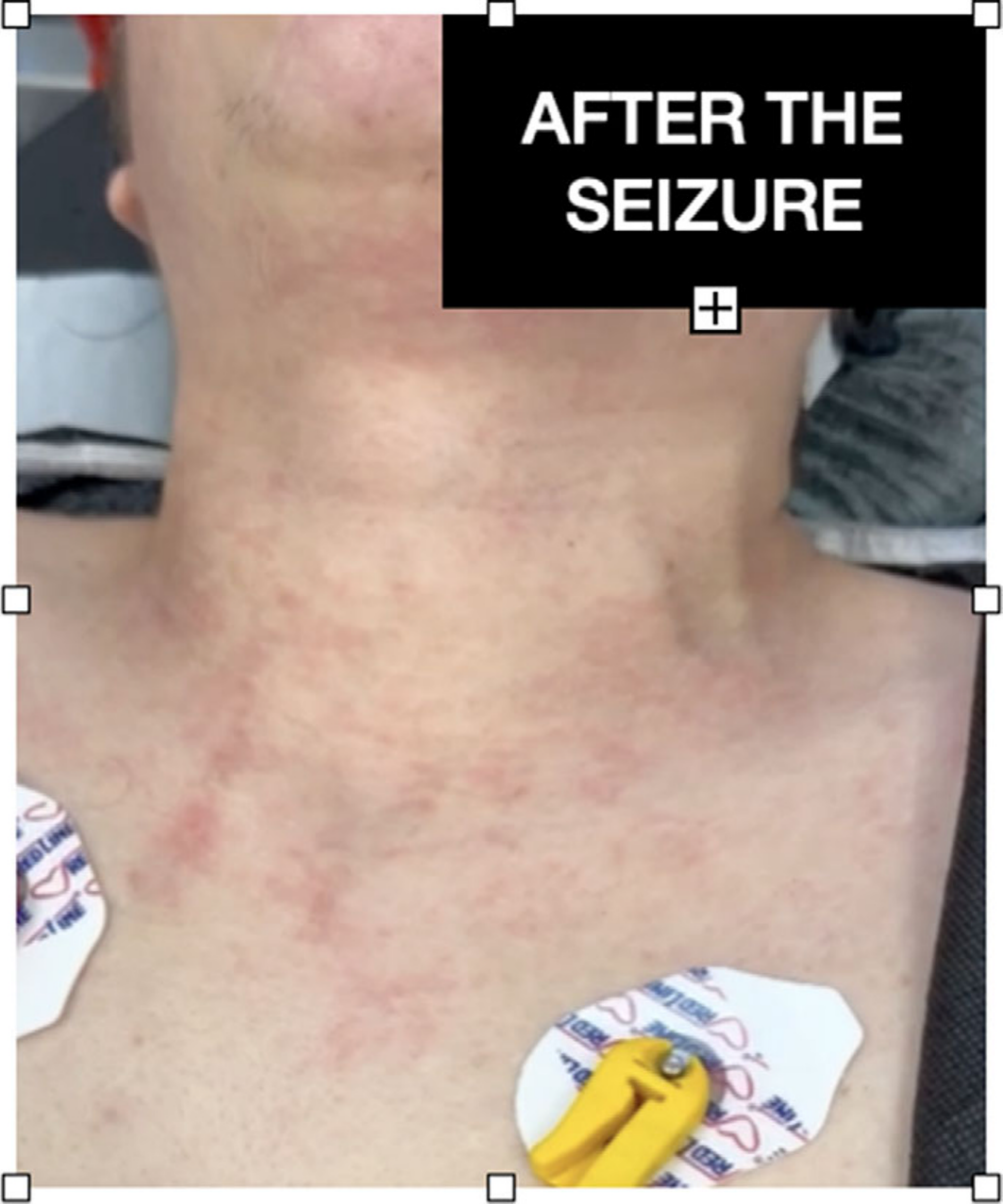

**Conclusions:**

This case illustrates a rare, energy-dependent dermatological reaction during bilateral ECT. Clinical features suggest a mast cell–mediated, IgE-independent process. ECT induces seizures via cortical and subcortical depolarization, which may activate autonomic pathways and trigger mast cell degranulation via neuropeptides (e.g.,substance P,calcitonin gene-related peptide) (Fetahovic et al.,2025). Mast cell mediators increase vascular permeability, causing erythema and edema (Voss et al.,2021). Rash severity correlated with ECT energy, not EEG/EMG seizure duration. The rash was transient, localized, and resolved rapidly with antihistamines and corticosteroids without anaphylaxis. ECT remained effective, improving psychotic symptoms.This case highlights a dose-dependent, manageable dermatological reaction and the neurostimulation–immune system interaction.

Consent: Written informed consent was obtained from the patient and her mother.

**Disclosure of Interest:**

None Declared

## Long descriptions

### Image 2: Long description

The table consists of four columns and seven numbered rows.

Column headers are: E C T Session, Energy Percentage (%), Seizure Duration on E E G / E M G (seconds), and Additional Medications For Rashes.

* Session 1: Energy 15 to 20; Seizure duration 14 to 20 / 11 to 8; Medication Prednisolone 40 mg.

* Session 2: Energy 30; Seizure duration 34 / 33; Medication Prednisolone 40 mg.

* Session 3: Energy 35; Seizure duration 33 / 39; Medication Prednisolone 40 mg.

* Session 4: Energy 40; Seizure duration 25 / 20; Medications Pheniramine Maleate 45,5 mg and Dexamethasone 8 mg.

* Session 5: Energy 45; Seizure duration 30 / 28; Medication Prednisolone 80 mg.

* Session 6: Energy 50; Seizure duration 25 / 25; Medication Prednisolone 80 mg.

* Session 7: Energy 55; Seizure duration 24 / 23; Medications Prednisolone 80 mg, Pheniramine Maleate 45,5 mg, and Dexamethasone 8 mg.

Navigate back to Image 2:.

